# Predominance of Human Bocavirus Genotype 1 and 3 in Outpatient Children with Diarrhea from Rural Communities in South Africa, 2017–2018

**DOI:** 10.3390/pathogens9040245

**Published:** 2020-03-25

**Authors:** Mpumelelo Casper Rikhotso, Ronewa Khumela, Jean Pierre Kabue, Afsatou Ndama Traoré-Hoffman, Natasha Potgieter

**Affiliations:** 1 Department of Microbiology, School of Mathematical and Natural Science, University of Venda, Thohoyandou 0950, South Africa; ronewakhumela@gmail.com (R.K.); kabuejeanpierre@yahoo.fr (J.P.K.); Afsatou.Traore@univen.ac.za (A.N.T.-H.); natasha.potgieter@univen.ac.za (N.P.); 2 Dean, School of Mathematical and Natural Sciences, University of Venda, Thohoyandou 0950, South Africa

**Keywords:** human bocavirus, acute gastroenteritis, rural communities, children

## Abstract

Human bocavirus (HBoV) is an emerging virus globally associated with diarrhea in young children. This study aims to investigate the prevalence of HBoV genotypes in children (≤5 years) from rural communities in South Africa (SA) suffering from acute gastroenteritis (AGE). A total of 141 fecal samples of children ≤5 years with acute gastroenteritis (AGE) were collected from rural primary health care facilities in the Vhembe district of SA between June 2017 and July 2018. Clinical symptoms and demographic data were also recorded. A total of 102 (72%) were outpatients, and 39 (28%) were hospitalized patients. Human bocavirus (HBoV) genotypes were determined using real-time multiplex PCR. DNA extracts of positive samples were confirmed by conventional PCR targeting the NS1 gene. Co-infection with other enteric viruses were determined in HBoV-positive samples using real-time PCR. HBoV was detected in eight (5.7%) children with AGE, of which three (37.5%) were HBoV1, three (37.5%) were HBoV3, and two (25%) were HBoV2. The majority of positive cases were identified in outpatients (62%) between the ages of 1 and 24 months. Co-infection in HBoV-positive samples with other enteric viruses included rotavirus (37.5%), adenovirus (37.5%), norovirus (25%), and astrovirus (12.5%). HBoV infections could be seen as a potential emerging diarrheal pathogen in South Africa. However, more studies are needed to understand the role of HBoV infections in children with AGE.

## 1. Introduction

Acute gastroenteritis (AGE) is recognized as one of the major causes of mortality in children ≤5 years of age in Africa and other developing countries [1,2]. AGE can be caused by several viral pathogens, including human bocavirus (HBoV), which is an emerging viral agent reported as a potential cause of diarrhea, especially in young children [3,4].

HBoV is a member of the Parvoviriadae family, Parvovirinae subfamily, and the genus Bocavirus [4]. Human bocavirus are small, nonenveloped, icosahedral viruses with an approximately 5.3 kb single-stranded DNA genome containing three open reading frames. (ORFs): the first ORF encodes NS1, the second ORF encodes NP1, and the third ORF encodes the viral proteins VP1 and VP2 [5]. There are currently four bocavirus genotypes identified globally, namely human bocavirus genotypes 1 to 4 [4,6]. 

HBoV investigations in South Africa (SA) have previously reported on the detection of HBoV genotypes in children with respiratory tract infections and AGE [7,8,9,10]. There is no published work on the detection of this virus from stools in children with AGE or children living in rural communities with poor water and sanitation infrastructure in South Africa. This study aimed to determine the prevalence and genetic diversity of HBoV in children with AGE from rural communities in Limpopo, South Africa. 

## 2. Materials and Methods

### 2.1. Study Design

The cross-sectional study was carried out between June 2017 and July 2018. Stool samples were randomly collected from patients at different clinics (outpatients) and hospitals (inpatients) from rural communities in the Vhembe region, Limpopo province of South Africa. Clinics and hospitals were both chosen as collection points because most cases of AGE in South Africa are seen by primary health care centers (clinics). These are situated in rural communities where the clinic refers only severe cases of AGE (with dehydration) to the hospital, where they take over the treatment plan. In total, 14 clinics and three district hospitals were visited during the study period.

### 2.2. Ethical Clearance and Consent

The study protocol was reviewed, approved, and registered by the ethics committee at the University of Venda (Ref. SMNS/17/MBY/03). Ethical clearance was obtained from the provincial Department of Health (Limpopo), South Africa (Ref: 4/2/2). Written, informed consent was given to the parent/guardian of the child to grant permission before participation and the collection of a stool sample from the child.

### 2.3. Sampling

In total, 141 stool samples were collected from children ≤5 years of age with AGE, of which 102 stool specimens were collected from clinics and 39 from hospitals. Only children who fit the criteria for acute gastroenteritis (diarrhea/vomiting/fever/cramping/dehydration) were recruited. Samples were transported to the laboratory after collection at 4 °C and stored at −20 °C until further analysis. 

### 2.4. Data Collection

Personal information such as age and sex was collected from patients, including consultation details, parental status, family living conditions, water source, and type of latrine facility used at home. Clinical symptoms, including symptoms of fever, vomiting, cough, diarrhea, and dehydration were recorded.

### 2.5. DNA Extraction and Amplification

The Boom method was used for DNA extraction, as previously described [11]. The method is based on the lysing and nuclease inactivating properties of the chaotropic agent guanidinium thiocyanate, together with the nucleic acid-binding proprieties of silica particles. The extracted DNA was stored at −20 °C until further analysis.

A HBoV real-time PCR commercial kit was used to detect HBoV1-4 genotypes following the manufacturer’s instructions (Liferiver, Shanghai, China). Amplification reactions were performed in a volume of 40 µL containing 35 µL reaction mix, 0.4 µL enzyme mix, 1 µL internal control, and 4 µL extracted DNA according to the manufacturer’s instructions. The real-time m-PCR was performed using the Corbett Research Rotor-Gene 6000 with the following conditions: 2 min at 37 °C, 2 min at 94 °C and 40 cycles of 15 s at 93 °C, and 1 min 60 °C. Positive samples were confirmed by sequencing, targeting the NS1 partial sequence.

### 2.6. Genotype Analysis

All positive samples from the real-time PCR assay were subjected to subsequent PCR targeting the NS1-nonstructural protein gene using the primers listed in Table 1.

The TopTaq Master Mix Kit (Qiagen) was used for the amplification of all positive HBoV samples following the manufacturer’s instructions. Amplification reactions were performed in a volume of 50 µL containing 25 µL master mix, 5 µL each of primer F and R, 15 µL RNase-free water, and 5 µL DNA. Amplification conditions for HBoV1 were initial denaturation for 10 min at 94 °C and 35 cycles of amplification (94 °C for 1 min, 54 °C for 1 min, and 72 °C for 2 min); the expected product size was 354 bp. HBoV2/4 amplification was initiated with a denaturation step of 15 min at 95 °C followed by 40 cycles of 94 °C for 15 s, 53 °C for 30 s, and 72 °C for 1 min; the expected product size was 454 bp for HBoV2/4. Human bocavirus 2 and 4 used the same antisense primers devised by Kapoor et al. [12], which were unable to differentiate between HBoV2 and 4 genotypes. Therefore, genotypes were further confirmed through sequencing. The amplification of HBoV3 consisted of 1 step of 95 °C for 15 min followed by 45 cycles of 94 °C for 20 s, 52 °C for 20 s, and 72 °C for 40 s, followed by an extension step at 72 °C for 10 min; the expected product size for HBoV3 was 440 bp [13]. 

All PCR products were visualized on 2% agarose gel stained with ethidium bromide. All positive samples were sequenced. The Sanger DNA sequencing was performed on the ABI 3500XL Genetic Analyzer POP7TM (Thermo-Scientific). The nucleotide sequences were compared with those of the reference strains available in the NCBI GenBank using BLAST tool available at http://www.ncbi.nlm.nih.gov/blast and then analyzed for their respective genotypes. 

### 2.7. Co-Infection Viruses Detected 

Co-infection with other enteric viruses was determined using a CFX96 (Bio-Rad) real-time PCR. The Allplex gastrointestinal panel virus assays (Seegene Technologies Inc., California, USA) were used to determine the prevalence of other enteric viruses in the stool samples, following the manufacturer’s instructions.

## 3. Results 

### 3.1. Study Population Characteristics

A total of 141 children with AGE were recruited in this study, of which 66 (47%) were males and 75 (53%) were females. A total of 83 (59%) children were aged between 1 and 12 months, 36 (25%) children were aged between 13 and 24 months, 10 (7%) children were aged between 25 and 36 months, nine (6%) children were aged between 37 and 48 months, and three (2%) children were aged between 49 and 60 months. All the children presented with symptoms of diarrhea (141; 100%). Fever (48; 27%), vomiting (47; 27%), dehydration (28; 16%), respiratory tract infection (26; 15%), and abdominal pain (27; 15%) were other observed symptoms. The majority (128/144; 90%) of the participants used tap water at home, and 119 (84%) used pit latrines.

### 3.2. Detection and Genotyping of HBoV Isolates 

The general characteristics of HBoV-positive patients are summarized in Table 2. The prevalence of HBoV genotypes 1 to 4 in stool samples of children with AGE was determined using real-time PCR. In total, eight (5.7%) samples were found positive for human bocavirus. HBoV1 was detected in three female patients with a mean age of 9 months; HBoV2 was detected in two patients of which one was male and one female, with a mean age of 9.5 months; HBoV3 was detected in three patients (37%) of which two were female and one was male, with a mean age of 19 months. HBoV4 was not detected in any of the 141 patients. Five (62%) of the positive cases were from clinics, and three (37%) of the positive cases were from hospitals (Table 2). Genotypes were determined through Sanger DNA sequencing and confirmed by comparison with reference genotypes available in the NCBI GenBank using BLAST tool. HBoV Sequences from the current study were deposited into GenBank under accession numbers MN072357-MN072360, MN082386, and MN082387.

Human bocavirus genotypes 1 and 3 were each detected in three (37%) stool samples, and genotype 2 was detected in two (25%) stool samples (Table 2) of patients. Among the eight positive HBoV samples, co-infection with other enteric viruses was found in seven out of eight (87.5%) patients, while infection with HBoV alone was detected in one out eight (12.5%) of the HBoV-positive patients. Mixed infections with rotavirus (three out of eight patients; 37.5%); norovirus (two out of eight patients; 25%); adenovirus (three out eight patients; 37.5%) and astrovirus (one out of eight patients; 12.5%) were observed in this study population (Table 2).

General characteristics of the eight HBoV-positive patient’s symptoms observed in this study were as follows: Among the three HBoV1 patients, one child (33.3%) had diarrhea, two children (66.6%) presented with diarrhea, fever, abdominal pain, vomiting, dehydration, and one patient had respiratory symptoms (Table 2). In HBoV2 patients, both children (100%) had diarrhea, while only one child (50%) had diarrhea and fever (Table 2). All HBoV3 positive children had diarrhea (100%), from which only one child (33.3%) had diarrhea, fever, vomiting, dehydration, and abdominal pain (Table 2).

**Figure 1:**
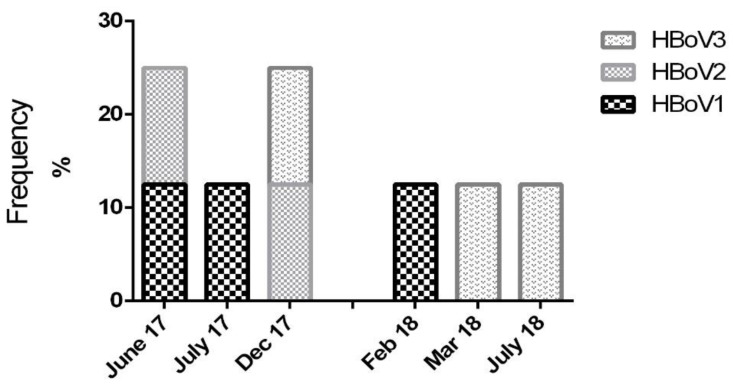
Human bocavirus genotype distribution (2017–2018).

HBoV 1 was observed in February (one out of eight patients; 12.5%), June (one out of eight patients; 12.5%), and July (one out of eight patients; 12.5%) (Figure 1). HBoV2 was only observed in June (one out of eight patients; 12.5%) and December (one out of eight patients; 12.5%), HBoV 3 was observed in March (one out of eight patients; 12.5%), July (one out of eight patients; 12.5%), and December (one out of eight patients; 12.5%) (Figure 1). 

## 4. Discussion

Several studies have reported human bocavirus in children with respiratory tract infections and AGE in South Africa [7,8,9,10]. However, no data are available on the prevalence of HBoV and other enteric pathogens in children with AGE from rural communities, suggesting that cases from these rural areas are most likely to be underinvestigated and under-reported [14,15]. This study, for the first time, investigated the prevalence of HBoV in children with AGE from rural communities in South Africa.

Altogether, a total of 141 stool samples from children with AGE were assessed for the presence of HBoV. The prevalence of 5.7% for HBoV in this study was comparable to other studies that have investigated HBoV in children with diarrhea worldwide [16,17,18]. Several of these studies have indicated that HBoV prevalence in patients with AGE ranges between 0.8% to 42% [16,17]. The high detection of HBoV in children from the clinics (outpatient) in the current study suggested that HBoV could be associated with mild to moderate diarrheal cases and asymptomatic cases [19]. Results from this study further suggest that HBoV could be seen as an emerging viral pathogen in the rural communities of South Africa. Although HBoV is considered a potential cause of diarrhea, available evidence supporting the causative role of the virus in acute gastroenteritis is inconclusive [20]. The presence of HBoV in asymptomatic individuals raises questions regarding the role of the virus in gastrointestinal infections as a pathogen or just as a bystander [20]. This is due to limited available studies investigating the pathogenesis of HBoV due to the lack of cell culture systems or animal models [12,21,22].

Reports worldwide have indicated that young children are prone to HBoV infections [23]. In this study, HBoV was detected in children between the ages of 1 and 24 months (≤24 months) (Table 2). No study to date has confirmed the association of HBoV infection with a specific age group of children affected. The virus could be infecting young children via fecal–oral-route transmission. A study in China [19] has reported that the transmission of HBoV was through ingestion of contaminated food/water (e.g., via flies, inadequate sanitation facilities, inadequate sewage and water treatment systems, and the cleaning of food with contaminated water), direct contact with infected feces (fecal–oral-route), person-to-person contact, and poor personal hygiene. In this study, the patients came from rural communities with no or inadequate water and sanitation infrastructure and poor hygiene practices [24]. The majority (128/144; 90%) of the participants used tap water in this study. From the eight positive cases of HBoV in this study, seven (87%) used tap water as a source of water, and only one (12%) used spring water as a source of water at home. Rural communities in South Africa usually collect water in storage tanks for both domestic and sanitation use due to scarcity of water in rural areas, and this results in the contamination of water through fecal–oral-route transmission [24]. Waterborne viruses represent a major health risk to the population worldwide [25]. There are currently limited data worldwide exploring the circulation of HBoV from environmental samples. Some studies have detected human bocavirus in river water [25,26] and wastewater samples [27,28,29]. Even though the role of HBoV in gastrointestinal infections is not fully understood, the risk of infection via contaminated water should be taken into consideration since many rural communities still face challenges of poor sanitation and hygiene practices [15]. In addition, the majority (119/144; 84%) of the participants in this study used pit latrines at home. All eight HBoV-positive cases used a pit latrine at home. The use of a pit latrine plays a role in the transmission of pathogens since the latrine lacks a handwashing facility and usually attracts flies which move between the facility and the house [24]. 

From the four known genotypes, only HBoV1, HBoV2, and HBoV3 were detected in this study. HBoV1 and HBoV3 were detected in three (37%) stool samples each, while HBoV2 was detected in two (25%) stool samples. Some studies have indicated that HBoV2, HBoV3, and HBoV4 are highly associated with gastroenteritis [30,31]. The widespread distribution of HBoV1 and HBoV3 in comparison to HBoV2 could presumably be due to differences in pathogenesis that may influence their transmission route and ability to establish persistence [32]. A study in Thailand [33] also only detected HBoV1, HBoV2, and HBoV3. Likewise, studies from Finland and Pakistan also detected genotypes HBoV1 to 3 [34,35]. In this study, HBoV4 was not detected. This was also the case in several other recent studies [6,34,36,37], which makes the role of HBoV4 genotype unclear. The predominance of HBoV1 and HBoV3 in children with AGE in this study is similar to a previous report in SA, which also observed a high prevalence of HBoV1 and HBoV3 [10]. However, the study only investigated HBoV in hospitalized children with AGE from an urban area, compared to this study which investigated both hospitalized and outpatients.

HBoV co-infection with other enteric viruses has been reported worldwide. In this study, HBoV was co-detected with other enteric viruses that are involved in acute gastroenteritis, including adenovirus F, which was detected in three out of eight (37.5%) samples. In comparison, rotavirus was detected in three (37.5%), norovirus in two (25%), and astrovirus in one (12.5%) out of eight samples. In only one out of eight (12.5%) of the human bocavirus positive samples, HBoV was detected alone without co-infection with other enteric viruses. A study in China [36] found HBoV co-infection with rotavirus was the most commonly detected (45.3%), followed by human coronavirus (10.1%), astrovirus (4.9%), and adenovirus (4.7%). Another study in Gabon [38] co-detected HBoV with rotavirus (33.3%), sapovirus (33.3), and adenovirus/norovirus (33.3%) in children with diarrhea. In this study, rotavirus (37.5%) and norovirus (37.5%) were the most commonly detected, followed by adenovirus (25%), and astrovirus (12.5%). Previous reports have indicated that HBoV co-infection with other enteric viruses is common. Studies from China, Thailand, Japan, Brazil, and Pakistan also reported that co-infection was very high, while rotavirus and norovirus were the most predominant co-infections [17,33,39,40]. 

A study in China [36] suggested that differences in the prevalence of certain HBoV genotypes might be due to regional differences in viral epidemiology. Some studies have suggested that HBoV has a seasonal peak during the spring months, while other studies suggested that the winter months, the geographic location, and seasonality [41,42] play an important role in HBoV prevalence. In this study, samples were collected over a period of twelve months. However, samples were not available for collection during each calendar month. Therefore, overall HBoV prevalence was inconclusive concerning seasonal patterns.

Limitations of the study included the absence of a control group (asymptomatic cases). Another limitation was the small number of stool samples collected. Human bocavirus infections are increasingly being recognized globally as a newly emerging virus associated with diarrhea. Therefore, surveillance of HBoV is crucial to monitor the prevalence and to help understand the role of this virus in individuals with AGE. The involvement of HBoV in children with AGE from rural communities in South Africa is most likely to be underinvestigated and under-reported. To the best of our knowledge, this study was the first to report on the prevalence and genetic characterization of human bocavirus in children with AGE from rural communities in Limpopo, South Africa.

## Figures and Tables

**Table 1:** Primers for human bocavirus (HBoV) genotyping.

HBoV Genotype	Target Gene	Primer	Sequence (5–3)	Fragment Length (bp)	Reference
**HBoV-1**	NS1	188F 542R	GACCTCTGTAAGTACTATTAC CTCTGTGTTGACTGAATACAG	354	[4]
**HBoV-2/4**	NS1	HBoV2-sf1 HBoV2-sr1	AACAGATGGGCAAGCAGAAC AGGACAAAGGTCTCCAAGAGG	454	[12]
**HBoV-3**	NS1	P5P6	CAGAAGCATCGGAAGTGGGTGT ATGTGAGGCTTTATGCTGGCTGA	440	[13]

**Table 2:** Characteristics of children showing positive detection of HBoV in fecal specimens.

Stool Specimen Collected (n = 141)	Positive Cases (n = 8)	Sample Code	Age (Months)	Sex	Symptoms						HBoV C_T_ Values	HBoV Genotype	Co-Infection with Other Enteric Viruses
					Diarrhea	Fever	Vomiting	Dehydration	Abdominal Pain	Respiratory			
**Clinics** 102 (72%)	5 (62%)	18	8	Female	1 (12.5%)	-	-	-	-	-	29.94 C_T_	HBoV1	Rotavirus, Astrovirus
		26	5	Female	1 (12.5%)	-	-	-	-	-	33.46 C_T_	HBoV2	Rotavirus
		119	13	Female	1 (12.5%)	-	-	-	-	-	17.94 C_T_	HBoV3	Adenovirus F
		105	14	Male	1 (12.5%)	1 (25%)	-	-	-	-	38.63 C_T_	HBoV2	-
		268	24	Female	1 (12.5%)	-	-		-	-	31.01 C_T_	HBoV3	Adenovirus F
**Hospitals** 39 (28%)	3 (37%)	11	20	Male	1 (12.5%)	1 (25%)	1 (33.3%)	1 (33.3%)	1 (50%)	-	34.04 C_T_	HBoV3	Norovirus, Adenovirus F
		40	7	Female	1 (12.5%)	1 (25%)	1 (33.3%)	1 (33.3%)	-	1 (100%)	33.39 C_T_	HBoV1	Rotavirus
		55	12	Female	1 (12.5%)	1 (25%)	1 (33.3%)	1 (33.3%)	1 (50%)	-	8.06 C_T_	HBoV1	Norovirus GII

HBoV = Human Bocavirus; **C_T_** = Cycle threshold.
